# Csl2, a novel chimeric bacteriophage lysin to fight infections caused by *Streptococcus suis*, an emerging zoonotic pathogen

**DOI:** 10.1038/s41598-017-16736-0

**Published:** 2017-11-28

**Authors:** Roberto Vázquez, Mirian Domenech, Manuel Iglesias-Bexiga, Margarita Menéndez, Pedro García

**Affiliations:** 1 Departamento de Microbiología Molecular y Biología de las Infecciones, Centro de Investigaciones Biológicas, Consejo Superior de Investigaciones Científicas, Ramiro de Maeztu 9, 28040 Madrid, Spain; 2 CIBER de Enfermedades Respiratorias (CIBERES), Instituto de Salud Carlos III (ISCIII), Madrid, Spain; 3 Departamento de Química-Física Biológica, Instituto Química-Física Rocasolano, Consejo Superior de Investigaciones Científicas, Serrano 119, 28006 Madrid, Spain

## Abstract

*Streptococcus suis* is a Gram-positive bacterium that infects humans and various animals, causing human mortality rates ranging from 5 to 20%, as well as important losses for the swine industry. In addition, there is no effective vaccine for *S. suis* and isolates with increasing antibiotic multiresistance are emerging worldwide. Facing this situation, wild type or engineered bacteriophage lysins constitute a promising alternative to conventional antibiotics. In this study, we have constructed a new chimeric lysin, Csl2, by fusing the catalytic domain of Cpl-7 lysozyme to the CW_7 repeats of LySMP lysin from an *S. suis* phage. Csl2 efficiently kills different *S. suis* strains and shows noticeable activity against a few streptococci of the mitis group. Specifically, 15 µg/ml Csl2 killed 4.3 logs of *S. suis* serotype 2 S735 strain in 60 min, in a buffer containing 150 mM NaCl and 10 mM CaCl_2_, at pH 6.0. We have set up a protocol to form a good biofilm with the non-encapsulated *S. suis* mutant strain BD101, and the use of 30 µg/ml Csl2 was enough for dispersing such biofilms and reducing 1–2 logs the number of planktonic bacteria. *In vitro* results have been validated in an adult zebrafish model of infection.

## Introduction

Antibiotics have been used as the common practice to prevent and treat infectious diseases in human, veterinary and agricultural fields during the last decades. However, the overuse and misuse of antibiotics have favoured the selection of multiresistant bacteria, leading to an alarming situation for severe infections, some of them currently untreatable by available drugs^1^. The decline of new antibiotic approvals, partly because of the disengagement of “Big Pharma” from antibiotic research and development due to low return on investment since late 90s, has made the situation even more critical. In such scenario, the World Health Organization (WHO) has alerted that we are in a race against time, so new antibiotics and preventive strategies development have become a priority to fight the rise of multiresistant pathogens, which is currently viewed as one of the most important health challenges to face in the near future^2^.

*Streptococcus suis* is an emerging zoonotic, Gram-positive pathogen capable of causing septicemia, arthritis, endocarditis, pneumonia, and meningitis both in pigs and humans^3,4^. At least 35 different serotypes have been identified in *S. suis* based on capsular polysaccharides^5,6^. Of them, serotype 2 –considered the most virulent– is one of the most prevalent isolates recovered from diseased pigs worldwide^7–9^, whereas serotype 9 has been reported as the most frequent isolate in diseased pigs in Europe^8,10^. Isolates of both serotypes infect humans who work in close contact with pigs or their products, and several outbreaks and hundreds of cases associated with pigs or human infections have been reported^11,12^. Three factors highlight the relevance of *S. suis* as a pathogen: (1) the human mortality rate, in the range of 5−20%^13,14^; (2) the development of resistance to penicillin, gentamicin, macrolides, and tetracyclines reported worldwide for many *S. suis* isolates^15^; and (3) the lack of an effective vaccine for *S*. *suis*, which favours its rising presence in humans and hinders the treatment^16^. All these facts make the case for searching alternative therapeutic agents against this bacterial pathogen.

Phage endolysins (also called lysins) are being increasingly recognized as one of the best alternative weapons to combat multiresistant pathogens. Lysins hydrolyze the bacterial cell wall by breaking specific bonds of the peptidoglycan and, as a consequence, kill the bacteria rapidly upon contact, much faster than conventional antibiotics. They have other important advantages that can be summarized as follows: (a) lysins are, in general, very specific to the pathogen, which preserves the normal microbiome; (b) their bactericidal activity is independent of the bacterial physiological state; (c) lysins can act synergistically with conventional antibiotics and reduce the development of resistance to these drugs when administered concomitantly; (d) to date, no resistance mechanisms (or resistant phenotypes) have been detected, probably because these enzymes target an essential and well conserved structural component like the peptidoglycan; (e) no signs of toxicity have been noticed after lysin treatment of systemic infections in mice models; (f) the immune response induced by lysins apparently neither neutralizes their activity nor prevents their use to treat systemic infections^17^.

Several putative prophages from *S. suis* have been reported^18^ and two virulent phages (SMP and Ss1) have been isolated and characterized so far^9,19^. From this bacteriophage diversity, a few attempts for obtaining lysins active against *S. suis* and with amenable anti-infective properties have been conducted. The PlySs2 lysin, from a prophage of a serotype 2 *S. suis* strain, has an N-terminal cysteine-histidine aminopeptidase (CHAP; PF05257) catalytic domain and a C-terminal SH3 type 5 (PF08460) cell wall binding domain (CWBD)^20^. PlySs2 is active against a broad range of Gram-positive bacteria *in vitro* and *in vivo* ^21^. With identical modular composition, the Ply30 and Ly7917 lysins kill *S. suis* strains of various serotypes as well as *Streptococcus equi* subsp. *zooepidemicus*, and protect infected mice^22,23^. LySMP, the first endolysin described to kill *S. suis* when used as an antibacterial agent^9^, has a different modular architecture to the three previous ones. Namely, it harbours an *Amidase_5* (PF05382) domain and a glucosaminidase (PF01832) domain flanking a CWBD made up of two CW_7 repeats (PF08230). CW_7 repeats are common constituents of endolysins from phages infecting a variety of Gram-positive pathogens^24^, being Cpl-7, encoded by the pneumococcal phage Cp-7, the most studied example of CW_7-containing endolysins. Cpl-7 harbours three almost identical CW_7 repeats connected by six residue-long identical linkers. These CW_7 repeats are essential for activity^25^ and have been proposed to target a common structure of the peptidoglycan network^26^.

The modular architecture of endolysins has allowed the successful swapping of functional domains to construct new chimeric proteins, some of them being even improved versions of the parental enzymes^27–30^. In particular, Cpl-711 chimera turned out to be the most lethal lysin with muramidase activity against pneumococci described to date. In fact, the Cpl-7 catalytic domain, which belongs to the GH25 family of glycosyl hydrolases (PF01183), is present in such chimera. Thus, it has proved to be a highly efficient catalytic unit by comparing the lytic activities against *Streptococcus pneumoniae* of the wild type forms and chimeric constructions between Cpl-1 and Cpl-7 lysozymes^28^.

In this work we have constructed a new chimeric lysin, Csl2, targeted to the zoonotic pathogen *S. suis*, by fusing the efficient catalytic domain of Cpl-7 and the two CW_7 repeats of the LySMP lysin. The Csl2 chimera has shown a strong bactericidal activity against certain strains of *S. suis*, from serotypes 2 and 9 and unencapsulated BD101 strain, and in a lesser extent against a few other streptococcal species of the mitis group. Csl2 activity was confirmed on planktonic cultures at different phases of the growth curve and biofilms, and validated *in vivo* using an adult zebrafish model of infection.

## Results

### Occurrence of CW_7 motifs and construction of the chimeric Csl2 lysin

**Figure 1:**
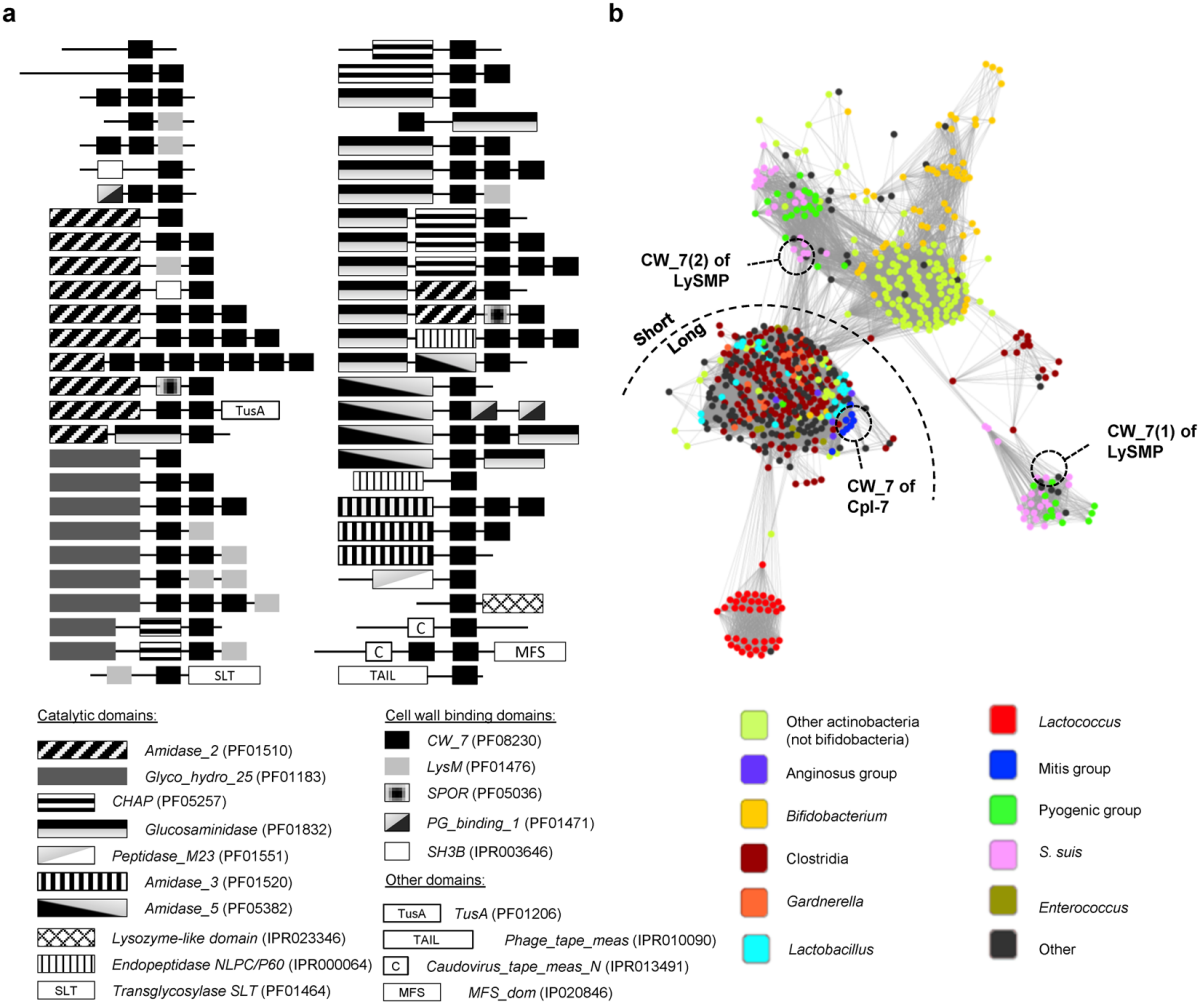
Occurrence and variability of CW_7 repeats. (**a**) Schematic representation of protein architectures containing CW_7 repeats. All possible architectures taken from the InterPro database are depicted, omitting redundant architecture entries. Comprised modules are pattern-coded and their designations according to InterPro or Pfam databases are shown at the bottom. Matching proteins can be accessed at https://www.ebi.ac.uk/interpro/entry/IPR013168?q=Cpl-7. **(b)** Sequence similarity network (SSN) of CW_7 repeats. Every node in the SSN represents a single CW_7 repeat found in InterPro database, edges are shown only when sequence identity between nodes ≥45%, and edges length are weighted regarding sequence identity. Nodes are coloured taking into account relevant taxonomic information, namely bacterial genera and other bacterial groups of interest.

**Figure 2:**
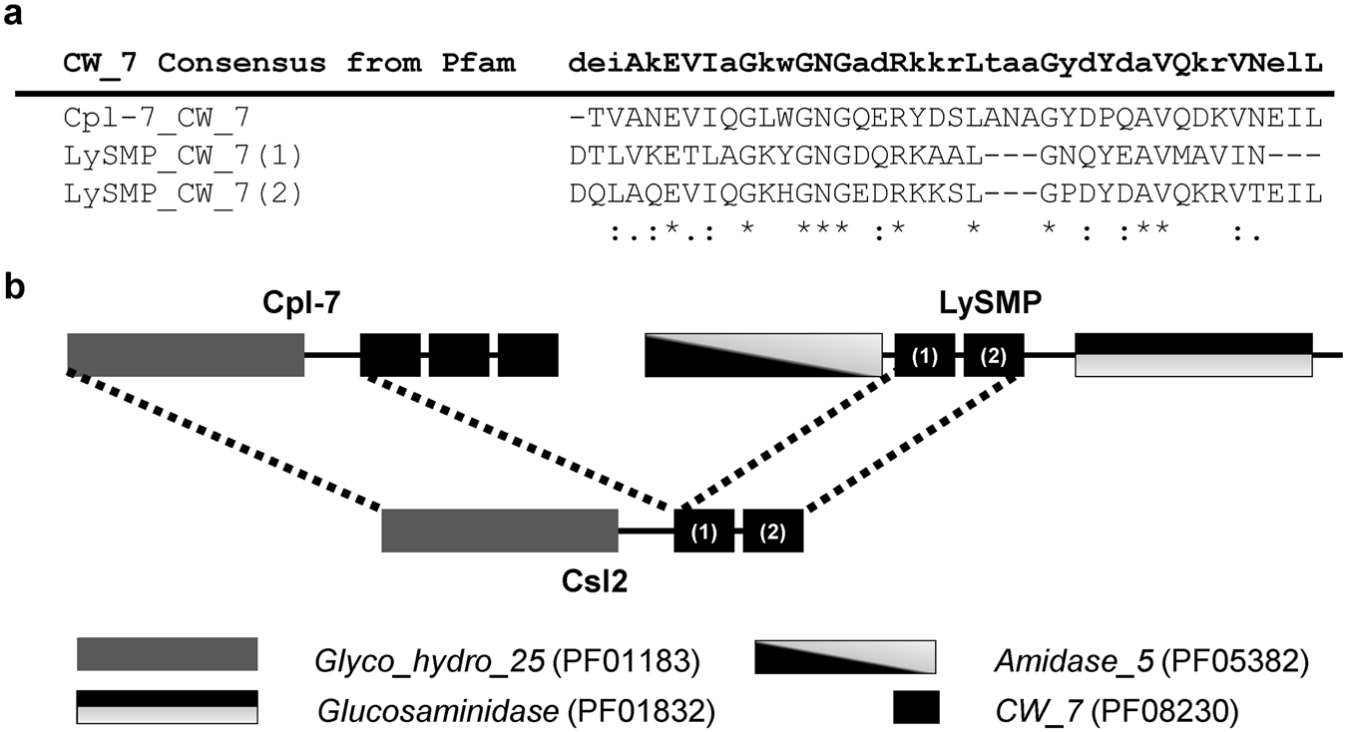
Csl2 chimeric lysin construction. (**a**) Sequence alignment of the CW_7 repeats found in Cpl-7 and LySMP lysins together with the consensus CW_7 sequence, taken from Pfam (PF08230). Strictly conserved residues (*) as well as conservative (:), and semi-conservative (.) substitutions within the alignment are indicated according to the automatic analysis performed with Clustal Omega. The most conserved residues in the CW_7 family are indicated in the consensus sequence in capital letters. **(b**) Schematic representation of the architecture of parental endolysins and the construction of the Csl2 chimera.

Analysis of databases has shown that genes encoding the CW_7 motif (PF08230; IPR013168) are mainly found in phages, either lytic or temperate. Such motifs can be forming part of functional virion genomes or located in bacterial chromosomes, either as active temperate phages or as remnants^24^. According to the InterPro database (last accessed, May 2017) there are 506 proteins containing CW_7 motifs –either as isolated motifs or together with other CWBD and/or catalytic domains– that can be grouped in 54 types of non-redundant protein architectures (Fig. 1a). The first described and characterized CW_7-containing protein was the Cpl-7 endolysin, which harbours a catalytic domain belonging to the GH25 family of glycosyl hydrolases^24,31^. The other two characterized lysins comprising CW_7 repeats are λSa2lys from a *Streptococcus agalactiae* prophage^32^ and LySMP from the *S. suis* SMP phage^9^, both sharing the same type of architecture [*Amidase*_5-(CW_7)_2_− glucosaminidase]. This means that most of the putative murein hydrolases that contain this kind of CWBD remain to be demonstrated to represent true enzymes. LySMP has a moderate to low activity on different *S. suis* strains and *Staphylococcus aureus*, and low activity against *S. equi* subsp. *zooepidemicus*, requiring reducing agents for full activity^9^. In contrast, the Cpl-7 lysozyme has an extended spectrum of bactericidal activity against streptococci, killing also *Enterococcus faecalis* and Gram-negative bacteria^33^, and does not require reducing conditions for activity. Moreover, its catalytic domain was shown to be extremely efficient when coupled to a high affinity CWBD^28^. As shown in Fig. 2a, the sequence identity percentages between the CW_7 repeats of LySMP [CW_7 (1) and CW_7 (2)] and that of Cpl-7 are 36% and 58%, respectively. LySMP and Cpl-7 repeats belong, respectively, to the short and long CW_7 subfamilies, which differ by three amino acid residues near the middle of the repeat^24^. This deletion is characteristic of the top peripheral similarity clusters of CW_7 repeats shown in Fig. 1b, whereas sequences in the central and bottom clusters are mostly of the long type. Moreover, the first and second repeats of LySMP are placed in two different clusters of the sequence similarity network that comprise almost exclusively sequences from *S. suis* and the pyogenic group.

Taking into account all these factors, we decided to construct a new chimeric lysin directed against *S. suis* by fusing the gene fragments encoding the catalytic domain and linker region of Cpl-7 and the tandem CW_7 repeats of LySMP. The rationale for this construction was: i) the potential high lethality of lysins carrying the catalytic domain of Cpl-7 lysin independently of redox conditions; ii) the presumed compatibility of this catalytic domain with CW_7 motifs; and iii) the possible adaptation of the CW_7 repeats of LySMP to the *S. suis* species. A schematic representation of the resulting Csl2 chimera and the parental proteins is shown in Fig. 2b.

### Biochemical characterization of Csl2

Details on the synthetic *csl2* gene, as well as cloning, overexpression and purification of Csl2 protein are included in Materials and Methods and Supplementary Figs. S1, S2. Csl2 is a bimodular protein of 287 amino acids (32.165 kDa) with an estimated net charge of −13.67 at pH 7.0 and a predicted p*I* of 4.72. The preparation yielded an average of 20 mg of Csl2 per liter of *Escherichia coli* culture with more than 95% purity, and a molecular mass of 32,021 Da (indicative of cleavage of the N-terminal methionine residue), as determined by SDS-PAGE and MALDI-TOF analyses (Supplementary Fig. S2). After purification, the native folding of the chimera was assessed by circular dichroism (CD). In the far-UV region, the CD spectrum of Csl2 closely resembles that of Cpl-7 (Supplementary Fig. S3), in agreement with their similar domain composition. The secondary structure contributions (33% α-helices, 17% β-sheets, 17% β-turns, and 33% of non-organized structure), estimated from the spectrum deconvolution, were also very close to previous CD-estimations for Cpl-7^24^ and consistent with the domain expected structures^24,34^. The near-UV spectra of Csl2 and Cpl-7 were also similar (Supplementary Fig. S3b), the difference in intensity being mainly attributable to the presence of either tyrosine [CW_7 (1)] or histidine [CW_7 (2)] in the repeats of Csl2 at the position occupied by a tryptophan in those of Cpl-7 (Fig. 2a)^26^.

**Figure 3:**
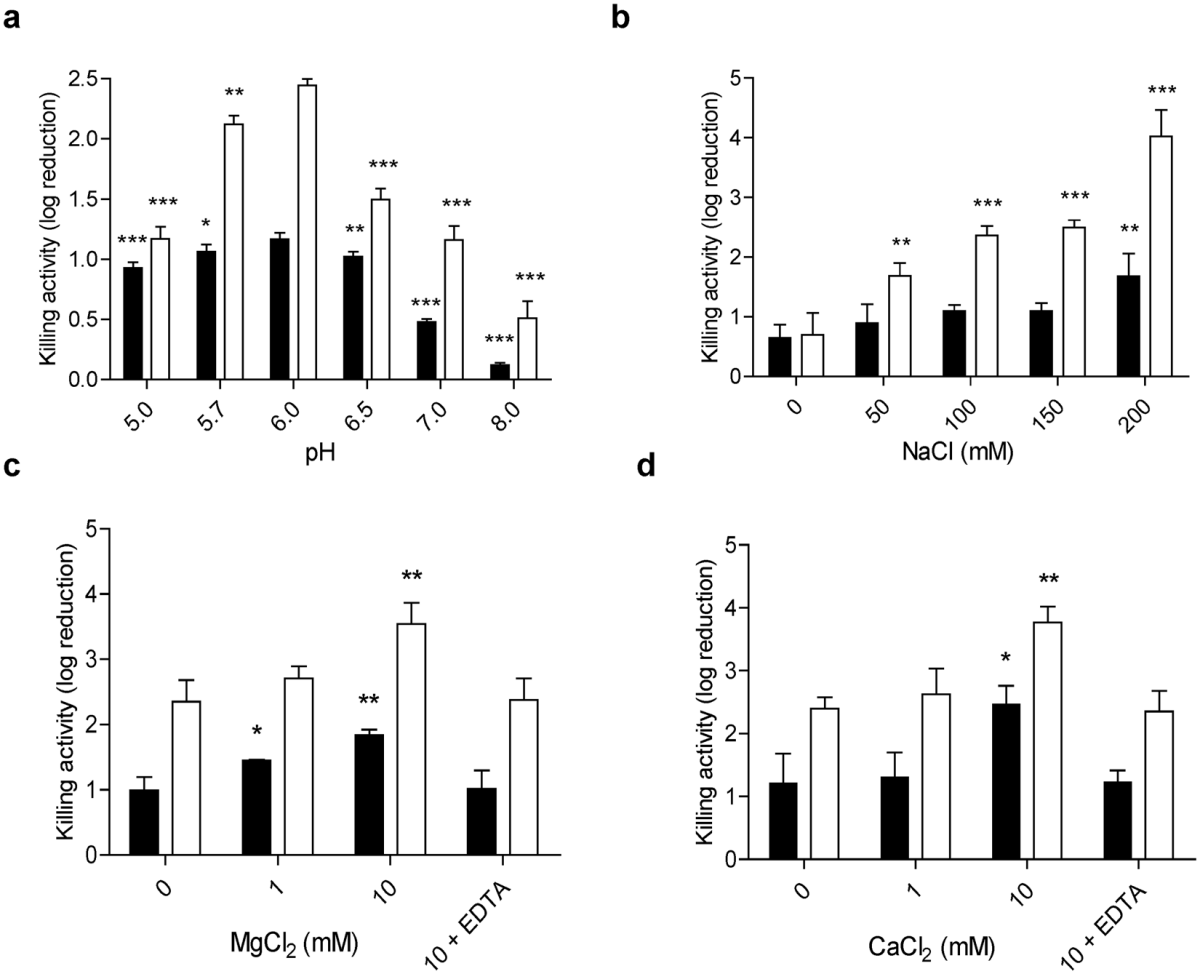
Biochemical characterization of Csl2 activity. Five μg/ml Csl2 were tested on *S. suis* 298 (black bars) or *S. suis* S735 (white bars) cultures resuspended in PB, pH 6.0, containing 150 mM NaCl with the indicated modifications. Incubation was continued for 60 min at 37 °C and bacterial cell counts were determined in blood agar plates after overnight incubation at 37 °C. (**a**) Effect of pH on Csl2 bactericidal activity. Acetate buffer was used for pH testing below 5.7. (**b** to **d**) Effect of NaCl, MgCl_2_ and CaCl_2_ concentrations on Csl2 bactericidal activity. The effect of Ca^2+^ and Mg^2+^ was reversed by adding equimolar concentrations of EDTA to the medium. Asterisks mean a significant difference (*P < 0.05; **P < 0.01; ***P < 0.01) according to one-way ANOVA followed by Dunnett post-test to compare with a reference value (pH 6.0 for panel **a** and 0 mM for panels **b** to **d**).

**Figure 4:**
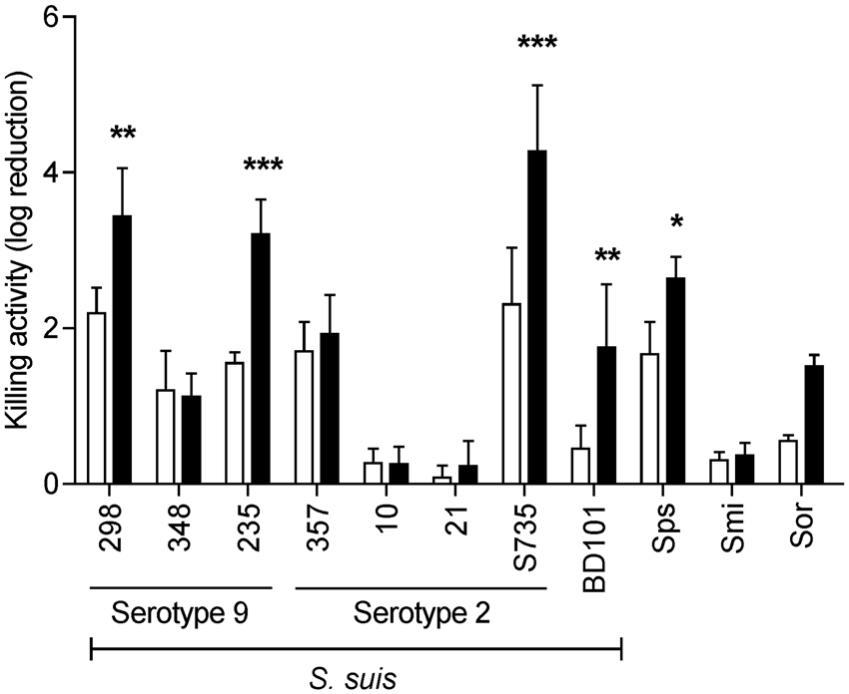
Effect of Ca^2+^ on Csl2 killing activity against a variety of *S. suis* strains and other susceptible streptococci. Activity assays were carried out with the addition of 15 µg/ml Csl2 on different bacterial cultures resuspended in 10 mM CaCl_2_ and 150 mM NaCl containing PB, pH 6.0 (black bars) or the same buffer without Ca^2+^ (white bars). Incubation was resumed for 60 min at 37 °C and bacterial cell counts were determined in blood agar plates after overnight incubation at 37 °C. Asterisks mark results in which Ca^2+^ had a statistically significant effect on Csl2 killing activity with respect to the control according to a two-way ANOVA test followed by Bonferroni post-test (*P < 0.05; **P < 0.01; ***P < 0.001). Sps, *S. pseudopneumoniae*; Smi, *S. mitis*; Sor, *S. oralis*.

Csl2 murein hydrolase activity was initially tested on radioactively labeled pneumococcal cell walls as described elsewhere^24^, showing some hydrolytic activity (5.7 × 10^3^ U/mg) but fewer than its parental enzyme Cpl-7 (1.1 × 10^5^ U/mg)^24^. Nonetheless, when tested on bacteria, Csl2 displayed no significant killing activity against *S. pneumoniae* although it was able to kill other streptococcal species, such as *S. suis* (see below). Therefore, the biochemical parameters for Csl2 optimal killing efficiency were tested using the *S. suis* type strain (S735, serotype 2) and 298 strain (serotype 9) as bacterial substrates, representatives of the most relevant serotypes described so far. The maximum bactericidal activity was observed between pH 5.7 and 6.5 (Fig. 3a), as it is common in lysozymes of the GH25 family, including Cpl-7 itself ^24^. The killing capacity of Csl2 was enhanced by increasing the ionic strength of the medium and/or by adding divalent cations (Fig. 3b–d), and the improvement of the activity was observed not only with the initial testing strains, but also with other susceptible *S. suis* strains and certain streptococci (Fig. 4). It is worth noting that the activity enhancement promoted by the presence of calcium and magnesium ions was reversed by the addition of equimolar concentrations of EDTA (Fig. 3d). Thus, the final standard buffer chosen to test the bactericidal activities was 20 mM sodium phosphate (PB), pH 6.0, containing 150 mM NaCl and 10 mM CaCl_2_. A similar behavior was reported by Ply700 lysin against *Streptococcus uberis* ^35^, although in this case the effect was only shown by turbidity reduction of bacterial suspensions. Regarding thermal stability, Csl2 kept full enzymatic activity after, at least, three weeks of storage at 4 °C or two weeks at 25 °C. In addition, Csl2 can be lyophilized without losing its bactericidal activity (Supplementary Fig. S4).

### Specificity of Csl2 bactericidal activity

The bactericidal activity of Csl2, as well as that of its parental Cpl-7 lysozyme, were tested on a variety of Gram-positive bacteria. The assayed bacteria included 8 strains of *S. suis*, 8 strains of other streptococcal species, and 2 strains from other genera. The summary of these results, shown in Fig. 4 and Table 1, revealed that the spectrum of bacteria killed by Csl2 was different than those of the parental enzymes Cpl-7 and LySMP^9,33^, being *S. suis* strains 298, 235 and S735 the most susceptible ones in the presence of Ca^2+^. It is worth to point out that the encapsulated serotype 2* S. suis* type strain (S735) was notably more susceptible to Csl2 than its isogenic non-encapsulated mutant BD101 (4-log killing versus 2-log when treated at 37 °C with 15 μg/ml Csl2 for 60 min in the Ca^2+^-containing buffer). Csl2 was also active against some streptococci from the mitis group, *e.g., Streptococcus oralis, Streptococcus pseudopneumoniae*, and *Streptococcus mitis* (Fig. 4 and Table 1). Remarkably, no significant bactericidal activity was observed against *S. pneumoniae, Streptococcus pyogenes* and *E. faecalis*, all of them efficiently killed by Cpl-7^33^, nor against *S. aureus*, which was slightly susceptible to LySMP^9^ (Table 1).

**Table 1:** Bactericidal activity spectrum of Csl2 and its parental enzyme Cpl-7.

Bacterial strain	Bactericidal activity	
	Csl2	Cpl-7
*S. pneumoniae* R6	NS	3*
*S. pseudopneumoniae* ^T^	2.6 ± 0.3	3.4 ± 0.5
*S. mitis* ^T^	0.4 ± 0.1	2.7 ± 0.6
*S. oralis* ^T^	1.4 ± 0.2	3.3 ± 0.3
*S. suis* S735	4.3 ± 0.9	3.1 ± 0.3
*S. mutans* ^T^	NS	0*
*S. dysgalactiae* sbsp. *equisimilis*	NS	1*
*S. pyogenes* ^T^	NS	3*
*S. iniae* ^T^	NS	1*
*E. faecalis* ^T^	NS	2*
*S. aureus* ^T^	NS	0*

Killing activity of 15 µg/ml Csl2 and 5 µg/ml Cpl-7 is shown in terms of log reduction (mean ± SD). NS indicates that no significant descent in viable counts was observed with respect to the control according to one-way ANOVA. Csl2 was tested in PB pH 6.0 with 150 mM NaCl and 10 mM CaCl_2_, Cpl-7 in the same buffer without calcium. Cpl-7 data marked with an asterisk (*) were taken from ref.^33^.

**Figure 5:**
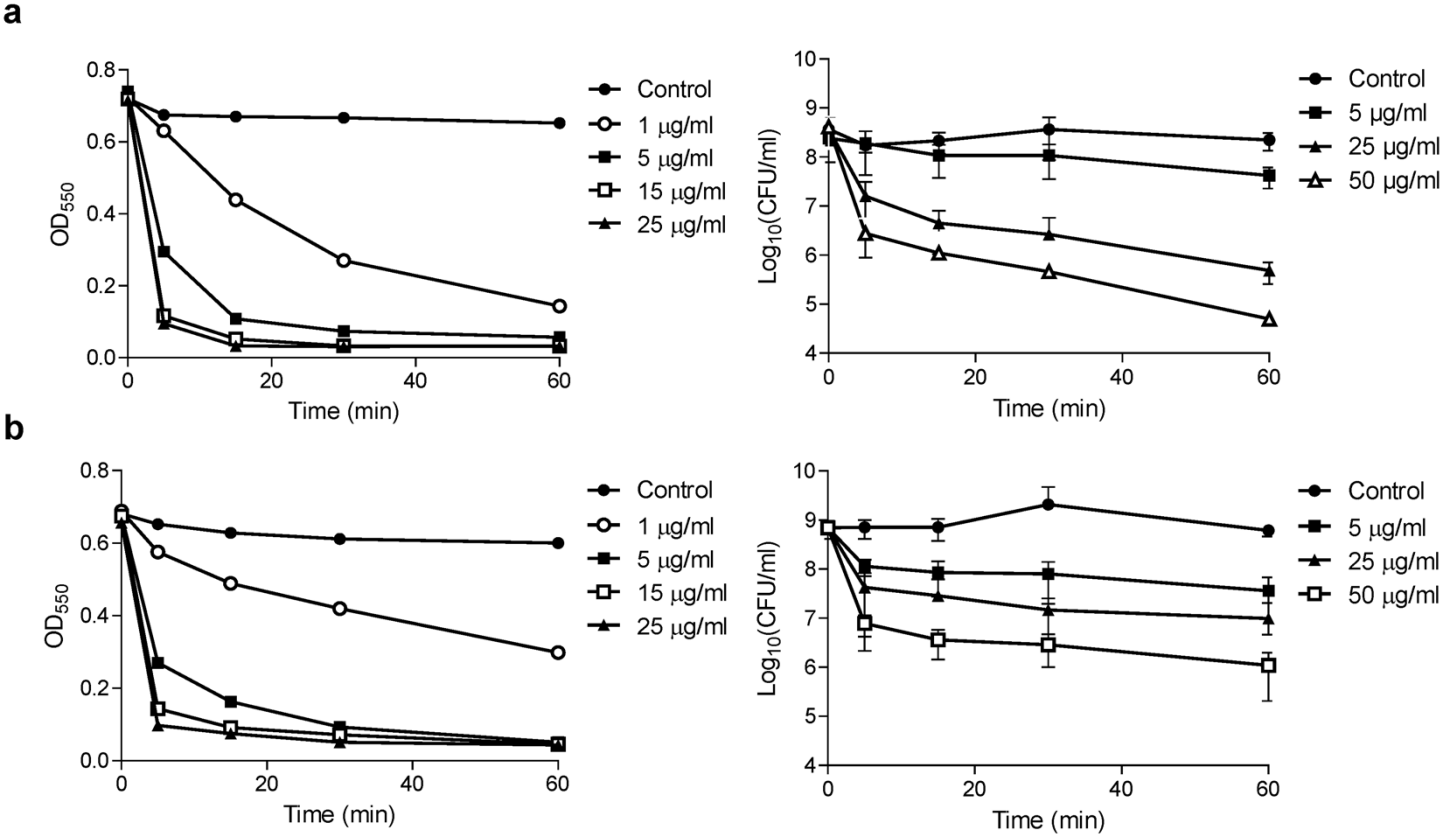
Dependence of Csl2 bacteriolytic and bactericidal activity on enzyme concentration. **(a)** Assays on *S. suis* 298 strain (serotype 9). **(b)** Assays on *S. suis* 357 strain (serotype 2). Turbidity decrease curves are representative results from three to five independent replicates, while viability curves represent means ± standard deviation. In order to observe a non-distorted bacteriolytic effect, the experiments were carried out at 37 °C in a non-calcium containing buffer (PB with 150 mM NaCl at pH 6.0).

The dose-response effect of Csl2 is illustrated in Fig. 5 where suspensions of *S. suis* strains 298 and 357 were exposed to a range of doses (1–50 μg/ml) as a function of time in the absence of Ca^2+^. Of note, Csl2 lethality reached a value of about 4 logs of viable cells when 50 µg/ml of the chimera were added to the *S. suis* 298 strain (serotype 9), whereas the same concentration of Csl2 killed 2.8 logs of *S. suis* 357, a serotype 2 strain. The pattern of the curves for both turbidity decrease and viable counts showed the rapid kinetics of *S. suis* 298 killing by Csl2, as maximal mortality ratio was almost achieved after 15–30 min of contact between the enzyme and the bacteria (Fig. 5).

### Csl2 activity against *S. suis* at different points of the growth curve

**Figure 6:**
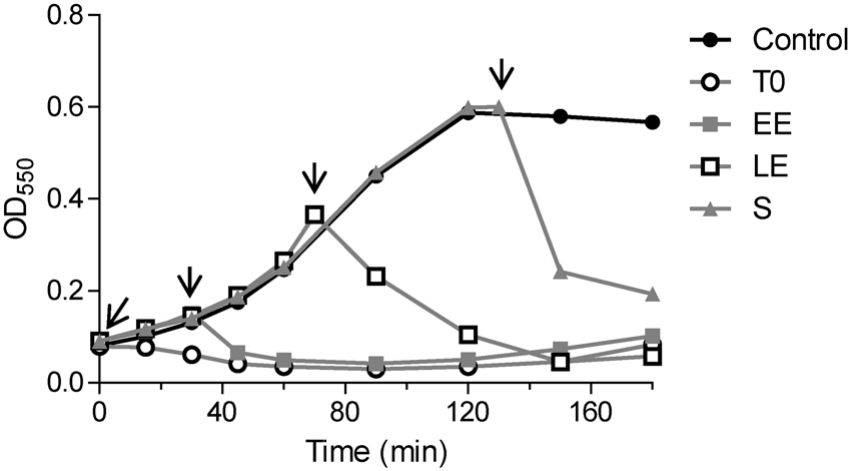
Bacteriolytic effect of Csl2 on different points of the growth curve. Turbidity evolution of growing *S. suis* 298 cultures is shown. Bacteria were grown in BHI and treated with 20 μg/ml Csl2 at the lag (T0), early exponential (EE), late exponential (LE), and stationary phases (S). The buffer-treated control culture is also displayed. Arrows indicate the enzyme addition times. Data are representative of three independent experiments.

To verify whether or not the bacteriolytic activity of Csl2 was maintained along the growing curve of the bacterial culture, we added 20 µg/ml of Csl2 at different points of the growth curve of *S. suis* 298 strain, *i.e*., lag phase, early exponential, mid-exponential, and stationary phases. The results clearly proved that this chimera displays a potent bacteriolytic effect in any growth phase, especially notable at the stationary phase, where a 63% reduction in OD_550_ was achieved in 20 min in a rich complex medium (BHI) (Fig. 6). When viable cells isolated from Csl2-treated cultures were subcultured in fresh medium and Csl2 was again added, the pattern of the new turbidity decrease curves and the bactericidal effects of Csl2 were identical to those of the original cultures (data not shown).

### Dispersing and lethal activity of Csl2 on *S. suis* biofilms

**Figure 7:**
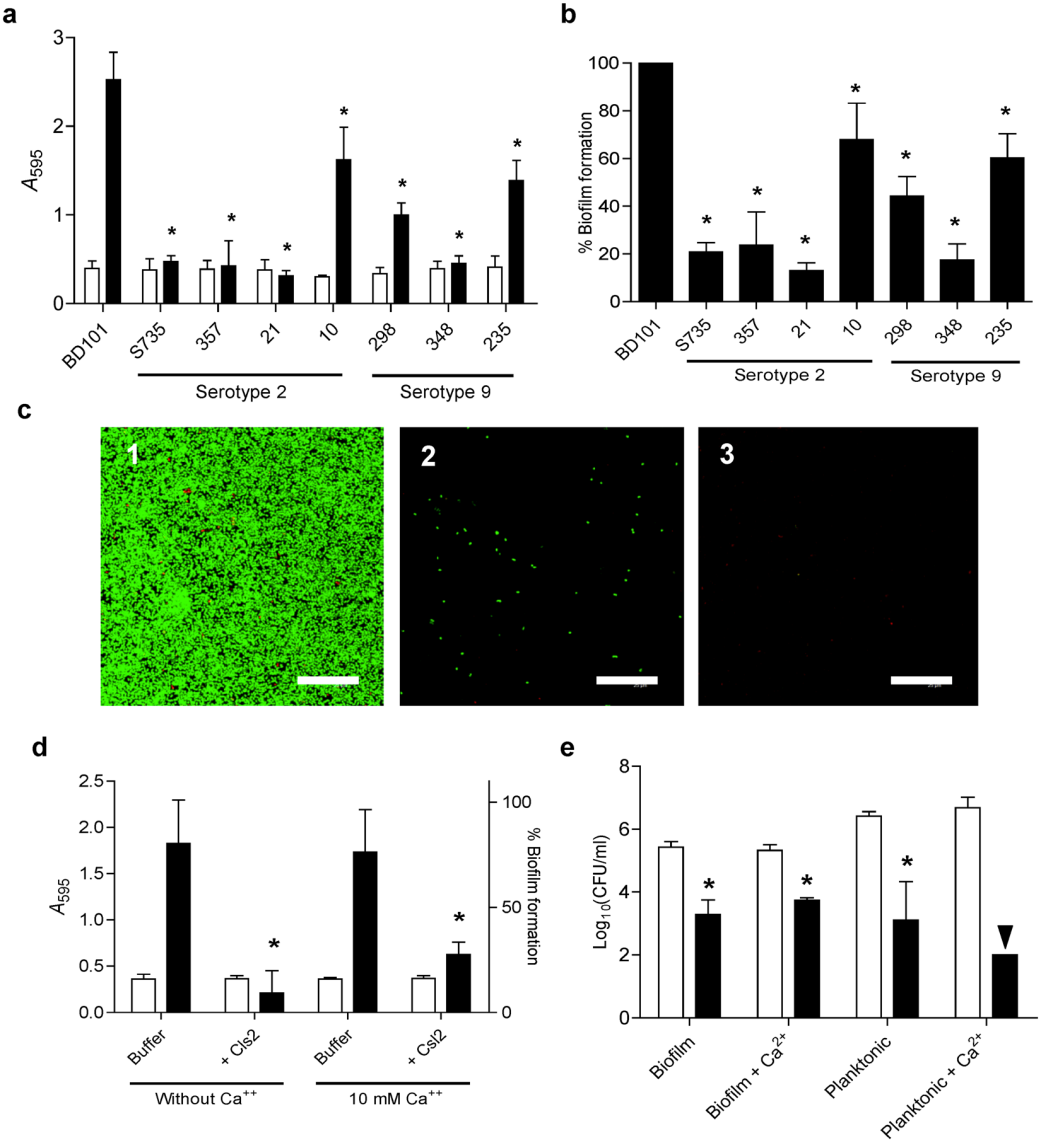
Biofilm formation capacity of different *S. suis* strains and antibiofilm activity of Csl2. **(a)** *A*_595_ measurements for CV stained biofilms of different *S. suis* strains. Conditions for biofilm formation were as described in Materials and Methods. White and black bars indicate growth (turbidity measurements of adherent plus non-adherent cells prior to CV staining as a growth control) and biofilm formation, respectively. (**b**) Relative biofilm formation capacity of capsulated strains compared to BD101 non-encapsulated strain. (**c**) CLSM image of the viability of biofilm-grown *S. suis* BD101 treated only with buffer (PB with 150 mM NaCl, 10 mM CaCl_2_, pH 6.0) (panel 1), with buffer containing 15 µg/ml Csl2 (panel 2) or with 30 µg/ml Csl2 (panel 3) for 1 h at 37 °C in the presence of 10 mM Ca^2+^. The biofilms were stained with the BacLight LIVE/DEAD kit to reveal viable (green fluorescence) and nonviable (red fluorescence) bacteria. Scale bars, 25 µm. (**d**) Csl2 antibiofilm activity quantification by CV staining. Biofilms formed by *S. suis* BD101 were incubated with buffer alone or buffer plus 30 µg/ml Csl2 for 1 h at 37 °C with or without 10 mM Ca^2+^. White bars indicate growth of adherent plus non-adherent cells (*A*_595_) and black bars indicate the percentage of biofilm remaining after buffer or enzyme treatment relative to the maximum biofilm formation. **(e)** Killing activity of Csl2 on *S. suis* biofilms. Viable countings were performed from samples of the biofilms and planktonic of the biofilms incubated with 30 µg/ml Csl2 (black bars) or buffer alone (white bars) at 37 °C for 1 h. The arrowhead (▼) means a value below detection limit (≤10^2^ CFU/ml). Statistical analysis for panels **a**, **b** and **d** was performed using a one-way ANOVA test followed by Dunnett post-test to compare with the non-encapsulated strain BD101 (panels** a** and **b**) or the respective control (panel **d**) (*P < 0.001); for panel **e**, **a** two-way ANOVA was performed followed by Bonferroni post-test (*P < 0.001).

It has been reported that the ability of *S. suis* to form biofilms is rather dependent on the strain, apparently varying with the capsular type or with the absence/presence of capsule^36,37^. This variability in biofilm formation capacity was in fact observed among the *S. suis* strains assayed in this work (Fig. 7a,b). Thus, as an *S. suis* biofilm model, we chose the non-encapsulated *S. suis* mutant strain BD101^36^, which was the best biofilm-forming *S. suis* strain of all tested. Quantification of the formed biofilm, by the classical crystal violet (CV) technique, yielded a maximum value of absorbance at 595 nm (*A*_595_) between 2–3 after 4–6 h incubation at 37 °C (Fig. 7a). Moreover, the BD101 biofilm was found to be a uniform layer, as confirmed by Confocal Laser Scanning Microscope (CLSM) (Fig. 7c), whereas the other *S. suis* strains tested failed to produce good enough biofilms in our experimental conditions. Once the biofilm was formed, Csl2 was added and the degree of disaggregation and killing of *S. suis* cells was tested. The results demonstrated that Csl2 disintegrated the matrix-embedded bacteria (Fig. 7d) but also reduced by around 2 logs the number of viable bacteria in the biofilm after 1 h incubation at 37 °C with respect to the control (Fig. 7e). The disintegration of Csl2-treated *S. suis* biofilms was also confirmed by CLSM (Fig. 7c). It is worth noting that the viable counts in the planktonics of the biofilms (*i.e*., the non-adhered cells present in the supernatant) were also reduced upon treatment with 30 μg/ml Csl2 with respect to the buffer-treated cultures by at least 2 logs, and even more when the buffer contained 10 mM Ca^2+^, since the number of viable cells was below the detection limit (Fig. 7c). This Ca^2+^-related increase in bacterial death in the planktonic cells of the culture correlates with the previously observed results with the BD101 parental strain S735 upon addition of 10 mM Ca^2+^ (Fig. 3d). Our data clearly revealed an improved anti-biofilm activity of Csl2 on *S. suis* in comparison to LySMP, which only showed a moderate anti-biofilm activity after 12 h-incubation time^37^.

### Activity of Csl2 in an adult zebrafish infection model

Zebrafish has been used for over a decade to study the mechanisms of a wide variety of inflammatory disorders and infections, with models ranging from bacterial, viral, to fungal pathogens. Zebrafish present adaptive immune response after 4–6 weeks. After this period, it is possible to carry out studies, as in the mammal models, to analyze novel antimicrobial agents^38^. Thus, in order to confirm *in vivo* the *in vitro* bactericidal activity of Csl2, we used an infection model of adult zebrafish in which bacteria were intraperitoneally injected^39–41^.

**Figure 8:**
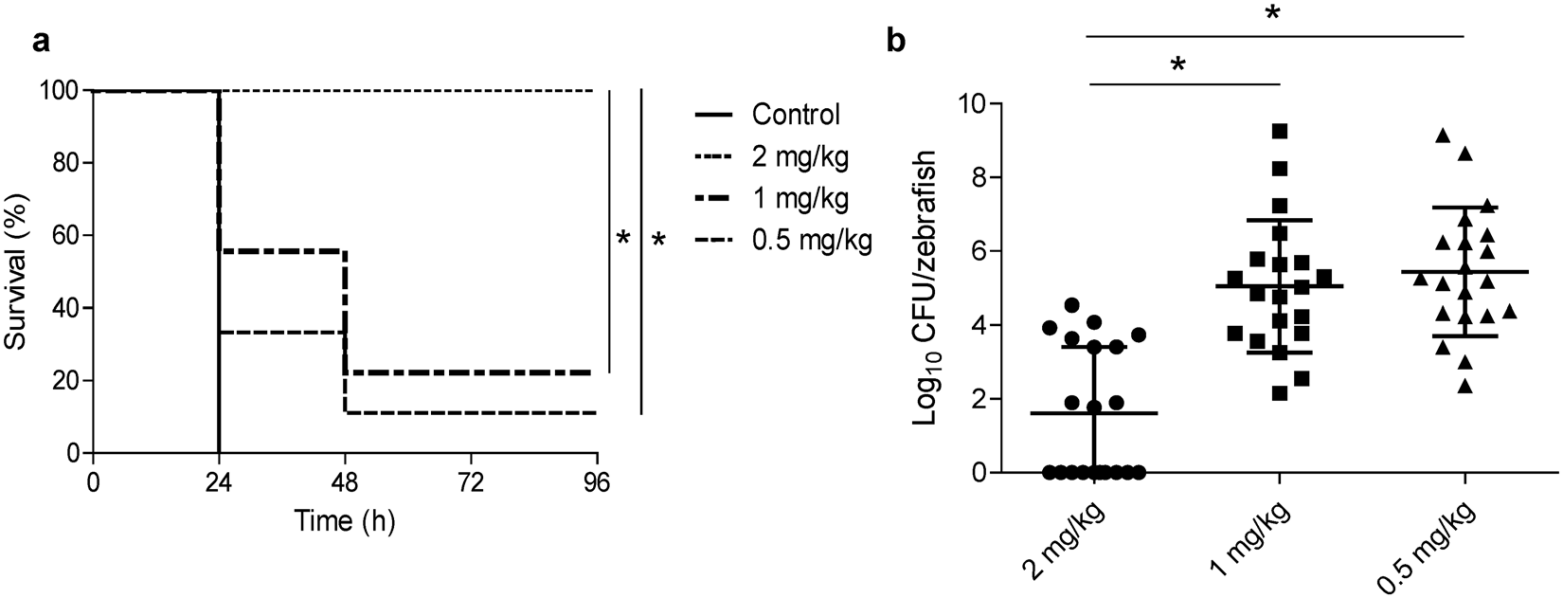
*S. suis* infection in the adult zebrafish model. (**a**) Survival of adult zebrafish infected with *S. suis* strain 298 and treated (or not) with Csl2 lysin (zebrafish: n = 20 per condition) is shown. Zebrafish were monitored for survival over a period of 4 days and results were plotted as Kaplan–Meier survival curves. Survival curves were compared with the log-rank (Mantel–Cox) and Gehan–Breslow–Wilcoxon tests (*P < 0.001). (**b**) Bacteria titers in the blood of zebrafish one day after challenged with *S. suis*. Blood was collected from the anal fin and the caudal fin in zebrafish, and the number of bacteria in the blood was determined. Each symbol represents the bacterial titer of a single zebrafish. Bars indicate the median bacterial titer. Statistical analysis was performed using an ANOVA test (*P < 0.01) followed by Dunnett post-test.

Zebrafish death provoked by the *S. suis* strain 298 infection (4.5 × 10^7^ CFU per zebrafish) occurred within the first day, and Csl2 injection 1 h after the bacterial challenge reduced the lethality resulting from such infection in a dose-depending manner. The level of protection increased from 11.2% (with 0.5 mg/kg) and 22.2% (with 1 mg/kg), to full protection at 2 mg/kg of Csl2 (Fig. 8a). Remarkably, the full protection achieved provided by Csl2 takes place with a drastic reduction of the bacterial load in the blood, in comparison with the groups treated with lower Csl2 concentrations. Twenty four h after infection, the colony forming unit (CFU) numbers in blood diminished down to 43 CFU per zebrafish, on average, in the group treated with 2 mg/kg of Csl2, with several zebrafish harbouring no viable bacteria, whereas zebrafish treated with either 1 mg/kg or 0.5 mg/kg displayed a mean CFU count of about 10^5^ CFU per zebrafish (Fig. 8b). This result indicates that bacterial growth in the blood was efficiently controlled by the Csl2 lysin.

## Discussion

In the last years it has become clear that one of the most effective strategies to fight bacterial pathogens, particularly the multiresistant ones, may arise from the phages that infect and lyse the host cells. This has led to a new era of phage therapy^42^. The evolutionary arms race between bacteria and phages for millions of years has optimized the mechanisms for rapid host lysis once the virions are complete and ready to spread. In many cases, the key factor to successfully accomplish this event is the phage endolysin, a murein hydrolase that cleaves specific bonds of the bacterial peptidoglycan. From the pioneering work of V. Fischetti’s laboratory and other researchers, lysins, also called enzybiotics, have been used as purified proteins exogenously added to the cultures for *in vitro* and *in vivo* killing of bacterial pathogens^17,43,44^. Currently, some encouraging clinical trials of lysins are under way, and the first lysin specifically directed against methicillin-resistant *Staphylococcus aureus* is already in the market (Micreos’ Staphefekt SA.100). In most phages infecting Gram-positive hosts, endolysins are made up of two functional domains, allowing the possibility to engineer new enzymes to improve catalytic or stability properties and to modify the spectrum of susceptible bacteria^27,30,33^. To achieve this goal, it is important to consider the crucial difference in the way lysins access to the bacterial cell wall in the context of the phage infection cycle or when used as enzybiotics. In the latter case, the interaction between the enzyme and its substrate takes place from the outside, and properties related to lysin charge and size, in addition to optimization of catalytic and cell wall binding domains, have been demonstrated to play important roles^28,33,45^.

In this work we have succeeded in constructing a new chimeric lysin by fusing the well characterized catalytic domain of the Cpl-7 lysozyme and the two CW_7 repeats of the LySMP endolysin as CWBD. The resulting chimera, Csl2, displays a strong bactericidal activity against some *S. suis* strains, and moderate to low activity against a few other streptococcal species belonging to the mitis group, with the noticeable exception of being ineffective against *S. pneumoniae*. The lethality of Csl2 against *S. suis* parallels those of PlySs2^21^, Ly7917^23^ and the Art-240 artilysin^46^, and surpasses those of LySMP, Ply30, λSa2lys and the ClyR chimera^9,22,46,47^. However, it is less active against *S. suis* than the parental Cpl-7 (it requires three times more enzyme to reach nearly the same killing activity against the strain tested). Interestingly, Csl2 displays a narrow lytic spectrum, thereby facilitating the specific treatment of *S. suis* infections. With the exception of PlySs2^20^, this trait is shared by the lysins from *S. suis* phages so far characterized^9,22,23^ and does not correlate with their modular composition, since PlySs2, Ly7917 and Ply30 comprise a CHAP domain and an SH3b domain. Furthermore, both Cpl-7 and λSa2lys, which share with Csl2 the catalytic domain and the type of CWBD, respectively, have extended lytic ranges of activity^33,46^. The explanation for such behaviour would require a detailed knowledge of the interactions established between each lysin and the cell wall of susceptible pathogens, and might be related to the emergent properties of catalytic domain and CWBD combinations rather than to the specific characteristics of each isolated domain. However, the differences in Cpl-7 and Csl2 killing activity should arise solely from the CWBD, given that they share the same catalytic module and differ only in their CWBD sequence and structure. In this respect, it has been suggested that the CW_7 repeats of Cpl-7 might recognize some fragment of the peptidoglycan as its receptor on the bacterial surface^26^. The presence of only two CW_7 repeats, belonging to different clusters of the CW_7 sequence-similarity network than those of Cpl-7 (Fig. 1b), would likely restrict the types of peptidoglycan receptors recognized by the Csl2 chimera in comparison with Cpl-7.

In-depth knowledge of the interactions created between the cell wall on one side, and catalytic domains or CWBDs on the other, is a must for the rational design of lysins directed against specific pathogens. Nonetheless, the results of this work, together with previous data of other CW_7-containing enzymes^9,32,33,46^ provide some insights of relevance. In particular, the analysis of sequence similarity networks within the different types of catalytic domains or CWBDs, and the correlation of the similarity clusters with taxonomic information might facilitate the identification of suitable partners to be combined in tailor-made lysins, directed against specific pathogens. Moreover, it might help to prevent the elimination of commensal organisms potentially susceptible to the action of lysins. In this regard, the CW_7 repeats have been also identified in the sequences of proteins encoded by normal microbiota^24^.

As for the biochemical properties of Csl2, it is noteworthy the boost of Csl2 bactericidal activity promoted by 10 mM CaCl_2_, reaching up to 4.3-log mortality in 60 min with strain S735 using 15 μg of Csl2 per ml (100-fold increase compared to the value obtained in calcium-free media). This enhancement was also confirmed in biofilm assays with the *S. suis* BD101 strain, where the number of viable planktonic bacteria in the biofilm supernatant was at least 10 times lower in calcium-containing than in calcium-free buffer. Remarkably, the presence of salts, either 10 mM CaCl_2_, 10 mM MgCl_2_ or 200 mM NaCl, reduced the turbidity decrease upon Csl2 addition, even though the number of viable cells decreased two or more log units (data not shown). This observation probably denotes a protective action against bacterial lysis while lethality was increased, which makes advisable to carry out both types of assays when measuring the effect of the medium composition on antibacterial activity, *i.e*. turbidity decrease and viable counting.

Current data strongly suggest that *S. suis* may contribute to the spread of antibiotic resistance genes to other streptococcal human pathogens, acting as a resistance reservoir^15^. Therefore, *S. suis* constitutes a paradigmatic example of possible intersections between animal and human resistomes. In addition, it is becoming clear that most persistent bacterial infections are associated with biofilm formation. This seems to be of particular importance in certain pathologies associated to *S. suis* infections like meningitis or endocarditis^48^. Interestingly, the isolation of naturally non-encapsulated strains is more frequent than previously thought, even in human cases of *S. suis* infection^49^, and their higher adhesive properties^48^ and propensity to form biofilms^36^ confer them advantages for bacterial colonization and the development of certain pathologies, including the aforementioned endocarditis. The lethality on *S. suis* biofilms makes specially promising a hypothetical application of Csl2 to fight *S. suis* infections. Indeed, Csl2 not only dispersed efficiently the bacteria that constitute the biofilm layer, but was also able to limit pathogen spreading by extensively killing the planktonic cells. This capacity, together with confirmation of Csl2 killing activity *in vivo* using the adult zebrafish infection model, strongly support the validity of our approach to design an efficient lysin targeted against this pathogen. Overall, current results reinforce the enzybiotic concept as a promising and effective alternative treatment to combat multiresistant bacterial pathogens.

## Materials and Methods

### Bacterial strains, media, and growth conditions

The bacterial strains and plasmids used in this study are listed in Supplementary Table S1. *E. coli* was grown in LB medium at 37 °C with shaking, *S. pneumoniae* R6 strain and *S. pseudopneumoniae* were grown without shaking in C medium supplemented with yeast extract 0.8 mg/ml (C + Y medium)^50^ at 37 °C, whereas *S. suis* and the rest of bacterial strains were cultured in BHI medium at 37 °C without shaking.

### Bioinformatic analysis of CW_7 repeats

All proteins containing annotated CW_7 domains (IPR013168) were retrieved from the InterPro database (last accession, May 2017). Such set comprised 506 distinct proteins containing 762 individual CW_7 repeats. With this set of unique repeats, a sequence similarity network (SSN) was constructed by using the Enzyme Similarity Tool from the Enzyme Function Initiative server (EFI-EST)^51^. Briefly, this web-based tool performs a local alignment using a BLAST-like algorithm from which every possible pair of sequences receives a score ×10^−x^, similar to the *E*-value obtained from a typical BLAST analysis. The construction of the SSN requires the definition of a cutoff score value. Below this threshold, sequence pairs are considered non-similar and, therefore, the pair will not be connected in the resulting representation of the SSN. In this particular case, the cutoff was set at 10^−11^, which, from our results, means that those sequence pairs whose similarity was below 45% were deemed non-similar (Fig. 1b). SSN was then graphically represented using Cytoscape 3.4^52^. The relevance and meaning of the clustered sequences were furthermore examined by feeding the graph with taxonomic information of the genomic origin of each individual repeat. In the case of CW_7 repeats from phage genomes, their taxonomic classification was assigned to the host organism of such phages.

### Cloning, expression, and purification of Csl2

The plasmids used in this work are shown in Supplementary Table S1. The chimeric *csl2* gene was chemically synthesized and purchased from ATG:Biosynthesis (Merzhausen, Germany) as an *E. coli* codon-optimized pUC-derived plasmid (pGHQ6). The NdeI–BamHI fragment carrying the chimeric gene was subcloned into the pT7-7 expression vector, previously cleaved with the same restriction enzymes, and the resulting recombinant plasmid (pT7Q651) was transformed into *E. coli* DH10B strain (Supplementary Fig. S1). For overproduction of Csl2, *E. coli* BL21(DE3) cells transformed with pT7Q651 were incubated in LB medium containing ampicillin (0.1 mg/ml) to an OD_600_ of ≈0.6. Isopropyl-β-d-thiogalactopyranoside (0.4 mM) was then added, and the incubation was continued for 4 h at 37 °C. Cells were harvested by centrifugation (15,000 × *g*, 20 min), resuspended in PB at pH 6.5, disrupted in a French pressure cell and centrifuged (20,000 × *g*, 20 min) at 4 °C to remove cell debris. Streptomycin sulfate (Sigma, 0.6 mg per mg of protein) was added to the crude extract and incubated for 20 min at 4 °C with slow stirring to precipitate DNA. The soluble fraction was loaded onto a DEAE-Sepharose column (HiTrap DEAE FF 5 ml, GE Healthcare) and a NaCl gradient, from 0 to 0.5 M, was applied (flow rate 1 ml/min). Csl2 eluted at ≈0.2 M NaCl and pooled fractions were subjected to a molecular exclusion chromatography (HiLoad 16/600 Superdex 200 pg column, GE Healthcare) in PB with 100 mM NaCl at pH 6.5 (flow rate 0.8 ml/min). Relevant Csl2-containing fractions and final purified sample were subjected to SDS-PAGE (Supplementary Fig. S2a). An aliquot corresponding to the Csl2 fraction of lane 3 was analyzed by MALDI-TOF to confirm the purity and molecular weight of the protein (Supplementary Fig. S2b). Csl2 concentrations were determined spectrophotometrically using the theoretical molar absorption coefficient at 280 nm (47,330 M^−1^/cm). Cpl-7 endolysin was purified and quantified as described^24^. Before use, all proteins were equilibrated by dialysis in the required buffer.

### Circular dichroism

Circular dichroism spectra were acquired at 25 °C in PB with 100 mM NaCl using a Jasco J-810 spectropolarimeter equipped with a temperature-controlled cell holder. Far-UV spectra were recorded from 260 to 200 nm in a 1-mm path length cuvette. Near-UV spectra were recorded from 320 nm to 250 nm in a 10-mm path-length cuvette. Csl2 concentrations of 0.2 mg/ml (far-UV) and 0.50 mg/ml (near-UV) were employed. Each CD spectrum was obtained by averaging 10 accumulations collected at a scan rate of 50 nm/min and 4 seconds of response time. PB spectra were subtracted from protein spectra and the corrected data were transformed into mean-residue molar ellipticities. Secondary structure composition was estimated by deconvolution of the far-UV spectrum with the CDNN program using reference data sets of 23 and 13 protein spectra^53^.

### Bacteriolytic and bactericidal assays

To measure the bacteriolytic and bactericidal activities of the enzymes, bacteria were grown to exponential growth phase (OD_550_ ≈ 0.3) and then centrifuged and washed with PB (pH 6.0) containing 150 mM NaCl, unless otherwise stated. The final OD_550_ was adjusted to 0.6 with the same buffer. Enzymatic activity was tested adding the enzyme to these bacterial suspensions in plastic tubes. Control experiments were run in parallel, substituting the enzyme volume by PB. Samples were incubated for 1 h at 37 °C and turbidity measures (OD_550_) were taken at selected intervals. Viability (CFU/ml) was determined in blood agar plates and colonies were counted after overnight incubation at 37 °C.

### Biofilm assays

*S. suis* biofilm cultures were obtained with a protocol similar to that described elsewhere^54^. Biofilm-forming strains were grown in C + Y medium to stationary phase (DO_550_ ≈ 0.6) and then conveniently diluted in the same medium to reach a cellular density of approximately 10^5^–10^6^ CFU/ml. Two hundred μl per well were added to a 96-well flat bottom polystyrene plate (Costar 3595; Corning) and subsequently incubated without shaking at 37 °C for 4–6 h. For determining the antibiofilm properties of Csl2, the supernatant containing *S. suis* planktonic cells was removed and substituted by 100 μl of the enzyme or the same volume of buffer (PB, pH 6.0, with 150 mM NaCl, adding 10 mM CaCl_2_ when specifically indicated) as control. Treated biofilms were then incubated for an additional hour at 37 °C. Biofilm formation and disaggregation was determined by CV staining: after incubation, *A*_595_ was measured to verify bacterial growth and, afterwards, 50 μl of a 1% CV solution were added to each of the wells. Staining of biofilms was allowed by incubating 15 min at room temperature. Following staining, biofilms were washed three times with distilled water and finally all liquid was removed and stained biofilms were resuspended in 200 μl ethanol for *A*_595_ measurement. The *A*_595_ was determined with a VersaMax microplate absorbance reader (Molecular Devices). The quantification of viable cells (separating planktonic and biofilm cells) was performed in blood agar plates.

For the observation of *S. suis* biofilms by CLSM, strains were grown on glass-bottomed dishes (WillCo-dish, WillCo Wells) for 4–6 h at 37 °C, plating 2 ml of inoculated C + Y medium on each dish. The biofilms were then treated either with 1 ml Ca^2+^-containing buffer alone or with Csl2 15–30 μg/ml for 1 h at 37 °C. Following incubation, the culture medium was removed and the biofilm rinsed with sterile water to remove non-adherent bacteria. Finally, they were stained with the bacterial viability BacLight kit (5 μM) (L7007, Invitrogen). After staining, the observations were made at a 63× magnification using a Leica TCS-SP2-AOBS-UV CLSM equipped with an argon ion laser. Images were analyzed using LCS software from Leica. Projections were obtained in the planes x–y (individual scans at 0.5 μm intervals).

### Ethics statement

Animal experiments conducted at The Zebrafish Lab (http://www.thezebrafishlab.com) were performed according to the European Union guidelines for handling of laboratory animals (http://ec.europa.eu/environment/chemicals/lab_animals/home_en.htm). Signs of infection were monitored three times daily throughout the experimental time course. Moribund zebrafish were euthanized by immersion in unbuffered MS222 solution (250 mg/l; 25–30 °C). Approval for these studies was granted by the University of Navarra’s Ethics Committee for Animal Experimentation (Protocol: 034-17). Experiments were carried out at The Zebrafish Lab’s animal housing facility with all efforts made to minimize animal suffering.

### Adult zebrafish infection assay

Zebrafish (n = 20 per condition) were injected with 10 μl of an *S. suis* strain 298 suspension (4.5 × 10^9^ CFU/ml), which was resuspended in perfusion solution (Grifols, Spain), and were incubated at 34 °C. One hour post–infection, individuals were divided into 4 treatment groups and were injected intraperitoneally with 10 μl of enzyme solution containing different doses of Csl2 (0.5, 1, and 2 mg/kg), or the perfusion solution as control. The survival rate for each experimental group was monitored three times per day for 4 days post infection. To further investigate the protective effect, the number of bacterial CFUs in blood in a preclinical sepsis model 24 h post treatment was determined. One day post-challenge, blood was collected from the caudal fin of zebrafish, and the number of bacteria in blood was determined. All infected zebrafish except those treated with Csl2 died before 24 h post infection and bacterial titer in their blood was not determined.

### Statistical analysis

All data are representative of results obtained from repeated independent experiments. Each value represents the mean ± standard deviation for at least 3 independent replicates unless otherwise stated. Statistical analysis was performed by using analysis of variance (ANOVA) followed by either Dunnett or Bonferroni *post hoc* tests. For *in vivo* data, the log-rank (Mantel–Cox) and Gehan–Breslow–Wilcoxon tests were used to draw, analyze and compare the survival curves. GraphPad InStat version 3.0 (GraphPad Software, San Diego, CA) was used for statistical analysis.

## Electronic supplementary material

Supplementary information
