# Supplementary material for: Csl2, a novel chimeric bacteriophage lysin to fight infections caused by Streptococcus suis, an emerging zoonotic pathogen
Source: Sci Rep. 2017 Nov 28;7:16506. doi: 10.1038/s41598-017-16736-0 (PMC5705598; doi:10.1038/s41598-017-16736-0)
Supplement: Supplementary file 1 — Supplementary information [file 41598_2017_16736_MOESM1_ESM.pdf]

Supplementary information

Csl2, a novel chimeric bacteriophage lysin to fight infections caused by *Streptococcus suis*, an emerging zoonotic pathogen

Roberto Vázquez[1,2], Mirian Domenech[1,2], Manuel Iglesias-Bexiga[2,3], Margarita Menéndez[2,3*] & Pedro García[1,2*]

[1]Departamento de Microbiología Molecular y Biología de las Infecciones, Centro de Investigaciones Biológicas, Consejo Superior de Investigaciones Científicas, Ramiro de Maeztu 9, 28040, Madrid, Spain. [2]CIBER de Enfermedades Respiratorias (CIBERES), Instituto de Salud Carlos III (ISCIII), Madrid, Spain. [3]Departamento de Química-Física Biológica, Instituto Química-Física Rocasolano, Consejo Superior de Investigaciones Científicas, Serrano 119, 28006, Madrid, Spain. [*]Correspondence and request for materials should be addressed to M.M. (email: mmenendez@iqfr.csic.es) or P.G. (email: pgarcia@cib.csic.es)

```
WT   ATGGTTAAGAAAAATGATTTATTTGTAGACGTTGCAAGCCATCAAGGCTACGACATTTCAGGAATTTTA
      M  V  K  K  N  D  L  F  V  D  V  A  S  H  Q  G  Y  D  I  S  G  I  L
OP   ATGGTGAAAAAAAACGATCTGTTCGTGGATGTGGCGTCCCATCAAGGTTATGATATTAGCGGTATTCTT

WT   GAAGAAGCAGGGACAACAAACACAATTATTAAAGTGTCAGAAAGTACAAGCTATTTAAACCCTTGCTTG
      E  E  A  G  T  T  N  T  I  I  K  V  S  E  S  T  S  Y  L  N  P  C  L
OP   GAAGAAGCGGGAACCACCAATACGATTATCAAAGTGAGCGAGTCGACAAGCTATTTGAACCCCTGCCTC

WT   TCTGCTCAAGTGAGCCAGTCAAATCCTATCGGGTTTTATCATTTTGCTTGGTTTGGTGGAAATGAAGAA
      S  A  Q  V  S  Q  S  N  P  I  G  F  Y  H  F  A  W  F  G  G  N  E  E
OP   GCAAGTGAGCCAATCTAATCCCATCGGTTTTTACCACTTTGCGTGGTTTGGGGGCAACGAGGAATCAGC

WT   GAAGCAGAAGCAGAAGCACGCTATTTCCTTGATAACGTGCCTACACAAGTTAAATACCTTGTACTAGAT
      E  A  E  A  E  A  R  Y  F  L  D  N  V  P  T  Q  V  K  Y  L  V  L  D
OP   GAAGCCGAAGCCGAAGCCCGTTACTTTCTGGACAATGTTCCGACGCAAGTGAAATACCTGGTTCTGGAT

WT   TATGAAGACCATGCAAGCGCAAGCGTACAAAGAAACACTACCGCGTGCTTACGCTTTATGCAAATTATC
      Y  E  D  H  A  S  A  S  V  Q  R  N  T  T  A  C  L  R  F  M  Q  I  I
OP   TACGAGGACCATGCAAGCGCGTCAGTCCAACGTAATACGACAGCATGTCTGCGGTTTATGCAGATCATT

WT   GCAGAAGCTGGATATACACCTATTTATTATAGTTACAAACCGTTTACGCTTGATAATGTGGACTATCAG
      A  E  A  G  Y  T  P  I  Y  Y  S  Y  K  P  F  T  L  D  N  V  D  Y  Q
OP   GCGGAAGCTGGTTATACGCCCATCTATTATAGCTATAAACCGTTTACGCTGGATAACGTAGATTACCAG

WT   CAGATTTTAGCACAGTTCCCTAATTCTCTATGGATTGCAGGCTATGGCTTAAATGATGGTACAGCTAAC
      Q  I  L  A  Q  F  P  N  S  L  W  I  A  G  Y  G  L  N  D  G  T  A  N
OP   CAGATCCTGGCCCAGTTCCCGAATAGTTTATGGATTGCAGGATATGGTCTGAACGATGGGACAGCAAAC

WT   TTTGAATACTTTCCAAGCATGGACGGTATCAGATGGTGGCAATATTCTAGTAACCCGTTTGACAAGAAT
      F  E  Y  F  P  S  M  D  G  I  R  W  W  Q  Y  S  S  N  P  F  D  K  N
OP   TTCGAATACTTTCCGTCGATGGATGGCATCCGTTGGTGGCAATACTCAAGCAACCCATTTGATAAAAAT

WT   ATTGTACTGTTAGATGATGAGAAAGAAGATAATATAAACAATGAAAACACTCTAAAAAGCCTTGATACA
      I  V  L  L  D  D  E  K  E  D  N  I  N  N  E  N  T  L  K  S  L  D  T
OP   ATTGTCCTGCTCGACGATGAAAAAGAAGACAATATCAATAATGAAAACACCCTGAAATCGCTTGATACG

WT   TTGGTTAAAGAGACTCTTGCTGGAAAATACGGAAACGGAGATCAGCGGAAAGCAGCTCTTGGTAATCAA
      L  V  K  E  T  L  A  G  K  Y  G  N  G  D  Q  R  K  A  A  L  G  N  Q
OP   CTGGTTAAGGAGACGCTGGCCGGTAAATACGGTAATGGGGATCAGCGCAAGGCCGCTCTGGGTAACCAA

WT   TATGAGGCTGTCATGGCAGTCATCAATGGCAAAGCTACGGCACCTAAAAAGACTGTTGACCAACTGGCT
      Y  E  A  V  M  A  V  I  N  G  K  A  T  A  P  K  K  T  V  D  Q  L  A
OP   TATGAAGCAGTTATGGCCGTCATCAACGGGAAGGCTACCGCGCCTAAAAAAACCGTAGACCAGCTGGCC

WT   CAAGAAGTGATTCAGGGGAAACATGGCAACGGTGAAGACCGTAAAAAATCCCTTGGTCCTGACTATGAC
      Q  E  V  I  Q  G  K  H  G  N  G  E  D  R  K  K  S  L  G  P  D  Y  D
OP   CAGGAAGTGATCCAGGGCAAACATGGTAACGGCGAGGACCGTAAAAAGTCATTAGGGCCCGATTACGAC

WT   GCAGTTCAAAAACGTGTAACTGAAATCCTGCAA
      A  V  Q  K  R  V  T  E  I  L  Q
OP   GCCGTGCAAAAACGCGTTACGGAAATCCTGCAG
```

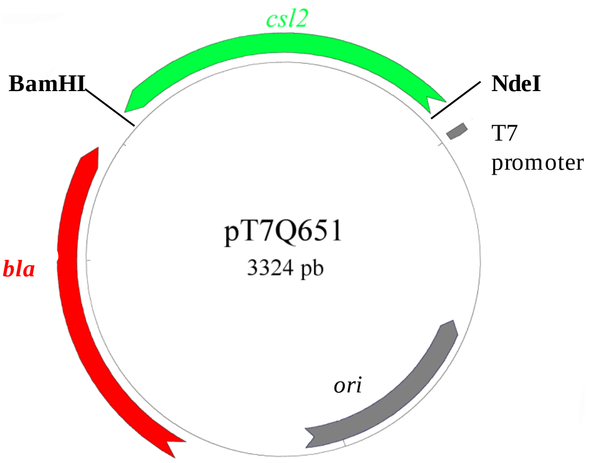

Supplementary Figure S1. Sequence of *csl2* chimeric gene and schematic representation of plasmid pT7Q651. Top, DNA sequences of the wild type (WT) and

codon-optimized (OP) gene fragments to construct *csl2* gene, together with the amino acid translation of such sequences. Nucleotides changed for codon optimization are highlighted in red and the linker region of Csl2 is underlined. Bottom, a scheme of recombinant plasmid pT7Q651 to express *csl2* gene, where the restriction enzymes used for cloning are indicated. *bla*, β-lactamase gene; *ori*, origin of replication.

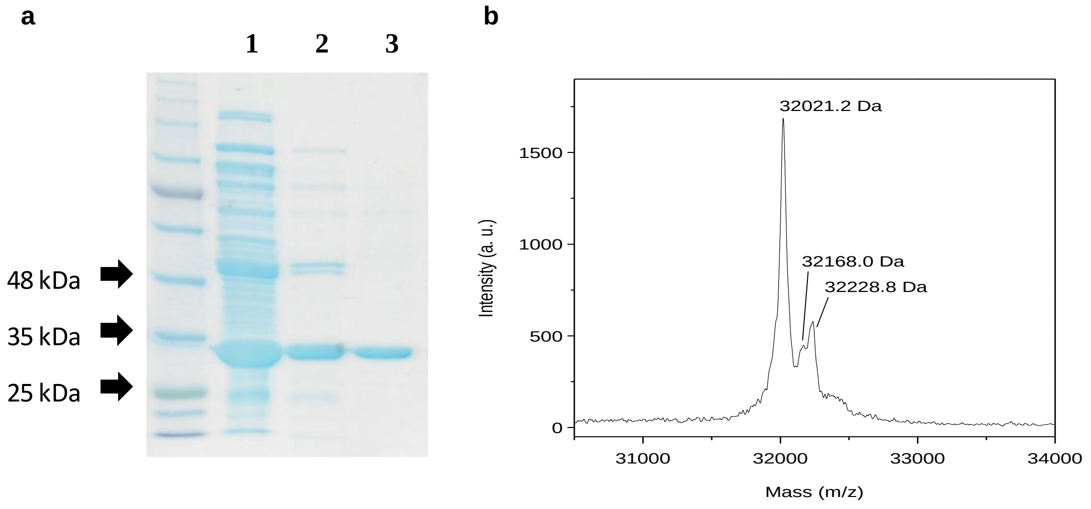

Supplementary Figure S2. Csl2 purification. (a) SDS-PAGE analysis of the Csl2-containing fractions. Lane 1, crude extract from BL21(pT7-7)(pT7Q651); lane 2, peak from DEAE-Sepharose column eluted at ≈ 0.2 M NaCl; lane 3, purified protein from HiLoad 16/600 Superdex column. Standard size markers, in kDa, are indicated on the left. **(b)** MALDI-TOF analysis of an aliquot of the sample loaded in lane 3 of the previous SDS-PAGE. The peaks at 32021.2 and 32228.8 correspond to the mass of Csl2 sequence with the initial methionine processed and its adduct with the MALDI-TOF matrix, respectively, whereas the small peak at 32168.0 would correspond to the non-processed sequence of Cls2.

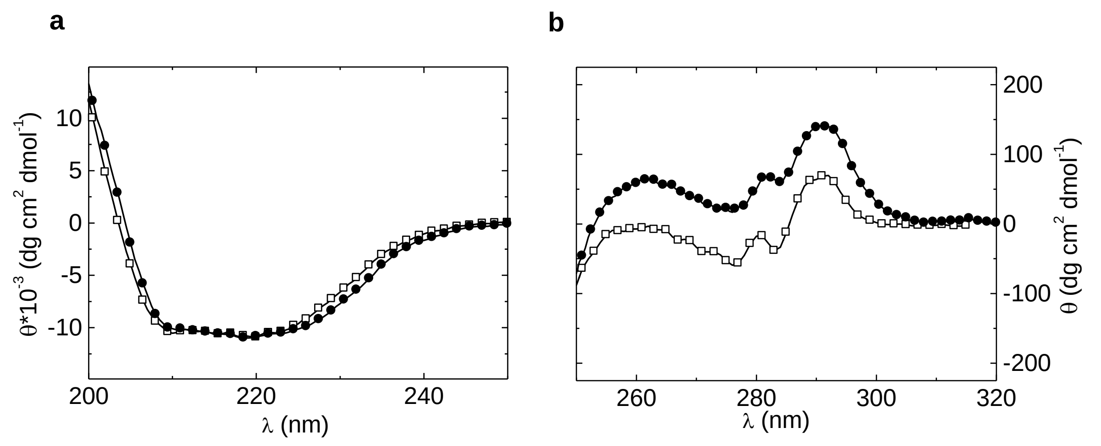

Supplementary Figure S3. CD spectra. Comparison of the **(a)** far- and **(b)** near-UV CD spectra of the chimera Csl2 (black circles) and Cpl-7 (white squares). Spectra were registered at 25 °C in PB (pH 6.5) with 100 mM NaCl and represented as mean residue ellipticities.

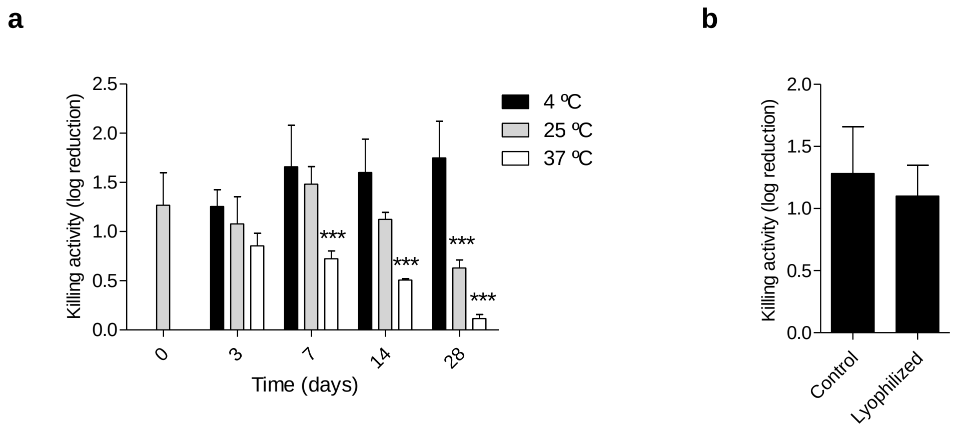

Supplementary Figure S4. Effect of temperature and lyophilization on Csl2 activity. (a) Purified Csl2 samples were incubated at the indicated times and temperatures. Then, 5 µg/ml of enzyme were tested on an *S. suis* 298 culture resuspended in PB, pH 6.0, containing 150 mM NaCl. Incubation was continued for 60 min at 37 ºC and bacterial cell counting was determined in blood agar plates after overnight incubation at 37 °C. ANOVA statistical tests were applied to verify that Csl2 samples kept at 4 ºC retained full bactericidal activity along time, whereas at 25 ºC and 37 ºC a significant reduction was observed when asterisk-marked (***, $p < 0.001$) **(b)** Bacteriolytic activity of 5 µg/ml purified Csl2 samples resuspended in PB, pH 6.0, containing 150 mM NaCl after lyophilization, together with that of the non-lyophilized control were checked as in **(a)**.

Strain/Name	Description	Source/Reference
Streptococcus suis		
298	Clinical isolate from pig, serotype 9	A. I. Vela
348	Clinical isolate from pig, serotype 9	A. I. Vela
235	Clinical isolate from pig, serotype 9	A. I. Vela
357	Clinical isolate from pig, serotype 2	A. I. Vela
10	Clinical isolate from pig, serotype 2	A. I. Vela
21	Clinical isolate from pig, serotype 2	A. I. Vela
S735	Type strain, serotype 2	M. Gottschalk
BD101	S735-derivative, non-encapsulated mutant	M. Gottschalk
Enterococcus faecalis	Type strain	ATCC 19433
Staphylococcus aureus	Type strain	ATCC 12600
Streptococcus dysgalactiae subsp. *equisimilis*		ATCC 9542
Streptococcus iniae	Type strain	ATCC 29178
Streptococcus mitis	Type strain	ATCC 49456
Streptococcus mutans	Type strain	ATCC 25175
Streptococcus oralis	Type strain	ATCC 35037
Streptococcus pyogenes	Type strain	ATCC 12344
Streptococcus pneumoniae R6	Non-encapsulated laboratory strain	1
Streptococcus pseudopneumoniae	Type strain	ATCC BAA-960
Escherichia coli		
DH10B	Bacterial host for cloning	2
BL21(DE3)	Bacterial host for protein expression	3
**Plasmids**		
pGHQ6	pUC derivative harboring synthetic gene *csl2*	This study
pT7-7	Expression vector, Ap^R	4

pT7Q651	pT7-7 derivative encoding *csl2*	This study

Supplementary Table S1. Bacterial strains and plasmids used in this study.

ATCC, American Type Culture Collection.

References

1. Hoskins, *et al.* Genome of the bacterium *Streptococcus pneumoniae* strain R6. *J. Bacteriol.* **183,** 5709–5717 (2001).

2. Durfee, T. *et al.* The complete genome sequence of *Escherichia coli* DH10B: insights into the biology of a laboratory workhorse. *J. Bacteriol.* **190,** 2597–2606 (2008).

3. Studier, F. W. Use of bacteriophage T7 lysozyme to improve an inducible T7 expression system. *J. Mol. Biol.* **219,** 37–44 (1991).

4. Tabor, S. Expression using the T7 RNA polymerase/promoter system, unit 16.2. *In: Curr. Protoc. Mol. Biol.* (Ausubel, F. M. *et al*. eds.). New York: Green Publishing Associates Inc. John Wiley & Sons (1990).
